# Executive function as a mediator in the link between single or complex trauma and posttraumatic stress in children and adolescents

**DOI:** 10.1007/s11136-017-1535-3

**Published:** 2017-03-11

**Authors:** Rosanne op den Kelder, Judith B. M. Ensink, Geertjan Overbeek, Marija Maric, Ramón J. L. Lindauer

**Affiliations:** 1 Research Institute of Child Development and Education, University of Amsterdam, Amsterdam, The Netherlands; 2 De Bascule, Academic Center for Child and Adolescent Psychiatry, Amsterdam, The Netherlands; 3 Department of Child and Adolescent Psychiatry, Academic Medical Center, University of Amsterdam, Amsterdam, The Netherlands; 4 Department of Developmental Psychology, University of Amsterdam, Amsterdam, The Netherlands

**Keywords:** Trauma, Executive functions, Posttraumatic stress, PTSD, Youth

## Abstract

**Purpose:**

In this study, we examined whether there is a mediating role of executive function (EF) in the relationship between trauma exposure and posttraumatic stress in youth.

**Methods:**

Children and adolescents exposed to trauma were recruited at an academic center for child psychiatry in The Netherlands. The total sample consisted of 119 children from 9 to 17 years old (*M* = 13.65, SD = 2.45). Based on retrospective life event information, the sample was divided into three groups: a single trauma group (*n* = 41), a complex trauma group (*n* = 38), and a control group that was not exposed to traumatic events (*n* = 40).

**Results:**

Our findings revealed that youth exposed to complex trauma had more deficits in EF compared to youth in the single trauma and control groups. EF was found to partly mediate posttraumatic stress symptoms for youth exposed to complex trauma, but not for youth exposed to single trauma. Youth exposed to complex trauma showed more deficits in EF, which was in turn associated with higher levels of posttraumatic stress symptoms.

**Conclusions:**

Our findings provide partial support for the role of EF in mediating posttraumatic stress outcomes for youth exposed to complex trauma. This points to the important role of EF in the etiology and treatment of complexly traumatized youth.

## Introduction

### Trauma exposure

Many youths experience a traumatic event before entering adulthood, with prevalence rates varying from 14 to 80% [1, 2]. According to the *Diagnostic and Statistical Manual of Mental Disorders*, a traumatic event is defined as one in which somebody experiences or witnesses a threat or violation of a person’s physical or psychological integrity [3]. As a result of exposure to traumatic events, youth may develop Post-Traumatic Stress Disorder (PTSD). PTSD symptoms are intrusive re-experiences (e.g., intrusive thoughts and nightmares), persistent avoidance (e.g., avoidance of feelings/thoughts related to traumatic events), negative alterations in cognitions and mood (e.g., feelings of detachment), and alterations in arousal and reactivity (e.g., sleep problems, hypervigilance) [3]. Youth diagnosed with PTSD experience academic, social, emotional, and physical problems [4].

### The role of executive functions in the development of PTSD

From a developmental perspective, exposure to traumatic events in childhood, when the brain is still developing, may impact neurological and cognitive development [5, 6], and thereby leave youth vulnerable to develop symptoms of PTSD. Specifically, executive functions (EFs) are hypothesized to be affected by trauma exposure and to play a role in the development of PTSD after trauma exposure.

Most studies in youth define EF as an umbrella term for separate, but related, cognitive processes [7, 8]. We describe EF as a range of mental skills that allow individuals to pay attention, manage their feelings, think in flexible and creative ways, control their impulses, plan and start activities, monitor their own performance, and remember and manipulate key information [9]. Three core concepts of EF are frequently addressed in empirical neuropsychological research in youth: inhibition, working memory, and cognitive flexibility [10, 11]. We consider these to be core concepts of a common EF factor from which higher order functions such as decision making and planning arise [11]. There are various outcomes associated with executive dysfunction in childhood and adolescence. For example, poor executive functioning has been associated with addictions [12], conduct disorders [13], obesity [14], poor treatment adherence [15], lower quality of life [16], and aggression [17]. In daily life, children with poor EF experience various difficulties: acting without thinking, overreaction to small problems, being upset by changes in plans, forgetting to hand in homework, delays in starting any kind of effortful task, switching between many tasks without finishing any, losing or misplacing things, difficulties meeting deadlines, difficulty setting personal goals, and lacking insight in their behavior [18].

### EF as a mediator in the link between trauma and PTSD

Results of a systematic review of adults with PTSD have shown that adults from 18 to 65 years perform significantly worse on EF measures than controls with other psychiatric disorders [19]. There is limited research on the association between trauma exposure and EF in youth, but some study results suggest that exposure to traumatic events can affect their EF. Familial trauma was related to poorer basic EF performance, compared to children exposed to non-familial trauma in a community sample [20]. Children exposed to maltreatment during multiple developmental phases performed lower on inhibitory control and working memory tasks than non-maltreated children or children that experienced maltreatment during one developmental period [21]. Maltreated youth also performed lower on cognitive flexibility than non-maltreated individuals [22].

EF could be a mediating factor in the association between trauma exposure and posttraumatic stress symptoms in children and adolescents. As trauma exposure negatively affects EF in youth [20–22], in turn, this could lead to posttraumatic stress symptoms. When emotion regulation or inhibitory control is decreased, they could have more difficulties inhibiting fear responses, intrusive thoughts, and experience more hypervigilance. While lacking the ability of inhibiting fear responses to triggers of the trauma, children and adolescents might develop an avoidant coping strategy [23]. The problems of hyperarousal, intrusions, and avoidance are core symptoms of PTSD.

Empirical evidence available indicates that trauma experience may impact EF differently in terms of timing and chronicity, which makes it important to make a distinction between single and complex trauma. Single trauma is defined as exposure to a single traumatic event, such as a traffic accident or rape. Children exposed to complex trauma have been exposed to multiple, persistent, and traumatic events (e.g., maltreatment, child sexual abuse, and neglect). Complex trauma is more often interpersonal, has an early onset, and more often occurs in the care-giving system of the child than single trauma [6]. Children with complex trauma histories develop more problems within various domains: attachment, neurobiological changes, affect regulation, dissociation, behavior control, and self-concept [24]. Moreover, results of a recent meta-analysis suggest that while approximately 16% of children exposed to trauma develop PTSD, the prevalence of PTSD in children differs greatly across single and complex trauma. Youth exposed to interpersonal trauma are 2.5 times more likely to develop PTSD than youth exposed to non-interpersonal trauma [25]. However, as previous studies have not made the distinction between single trauma and complex trauma, it remains unclear how trauma exposure impacts EF differently for children exposed to single or complex trauma. The current study helps closing this knowledge gap by giving more insight in the possible differential impact of single and complex trauma on EF. Clinical practice could also benefit from this study as we gain more knowledge about how exactly single and complex trauma are related to problems in EF in youth.

### Research questions

Drawing from the literature and theoretical framework [5], the following research question was devised: To what extent is EF a mediator in the relationship between trauma exposure and posttraumatic stress in youth? First, we hypothesized that there is a negative association between trauma exposure and EF in children and adolescents [20, 23]. Therefore, we compared youth exposed to traumatic events (both single and complex trauma) with healthy control youths that did not experience traumatic events. Considering that EF develops across childhood and adolescence [11], we hypothesized that EF is more likely to be impacted by complex trauma than by single trauma [21, 26, 27]. Third, we hypothesized that EF plays a mediating role in the relationship between complex trauma and posttraumatic stress in youth, but not in the relationship between single trauma and posttraumatic stress [23, 28].

## Method

### Sample

The current study compared EFs between children exposed to single trauma, exposed to complex trauma, and children that did not experience trauma in a cross-sectional research design. Twelve participants were excluded from our study because of missing screening questionnaires due to language barriers of parents, excessive loads on the parental burden, and unstable home environment with changing caregivers. The total sample consisted of 119 participants (65 girls) aged 9–17 years old (*M* = 13.65, SD = 2.45). The control group consisted of 40 children (17 girls) aged 9–17 years old (*M* = 13.88, SD = 2.50), the single trauma group consisted of 41 children (24 girls) aged 10–17 years old (*M* = 14.00, SD = 2.04), and the complex trauma group consisted of 38 children (24 girls) aged 9–17 years old (*M* = 13.03, SD = 2.73).

## Procedure

Our study was part of ongoing research on genetic and neurological vulnerability, including EF, in the development of PTSD in youth. For this study, we obtained permission from the Medical Ethical Committee of the Academic Medical Center in Amsterdam and the Ethics Committee of the University of Amsterdam, The Netherlands.

**Fig. 1:**
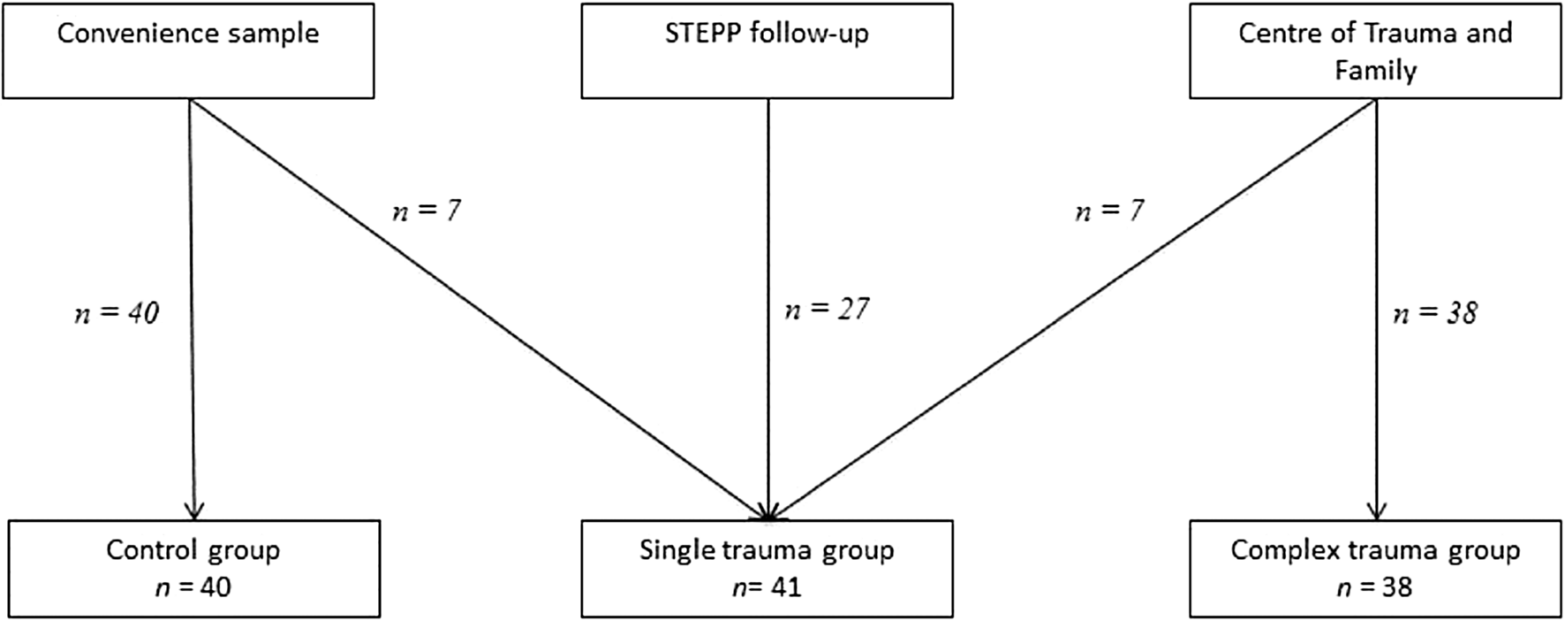
Flow diagram of participants

Recruitment differed between traumatized participants and the control group, and there were two lines of recruitment of children exposed to traumatic events (see Fig. 1). First, trauma-exposed children and adolescents were recruited during a follow-up of a research project of the Academic Medical Center of the University of Amsterdam that focused on PTSD in children who were involved in an accident [29]. Researchers contacted these participants and their caregivers during follow-up of this research project and asked them to participate in the current study. Second, youth exposed to traumatic events were recruited at the Center of Trauma and Family at De Bascule, Academic Center for Child and Adolescent Psychiatry in Amsterdam. Youth, aged 8–18 years, were recruited and assessed before the start of trauma treatment. Many studies that investigated complex trauma included treatment seeking individuals [e.g., 30, 31], because in complexly traumatized individuals treatment seeking is the norm rather than the exception. Individuals in a treatment setting were a logical group to recruit and are a representative sample for complex trauma. Researchers provided information about the study, its aims, and the research procedure. While informing them about the research, we highlighted that participation was voluntary and would not affect their possible treatment program. Regular intake procedure consisted (among other aspects) of assessment of trauma exposure and trauma symptoms and a parent questionnaire about EF. Children exposed to traumatic events were then subdivided into a single trauma and complex trauma group based on their retrospective information about trauma exposure. Children who were exposed to prolonged or recurrent traumatic events were assigned to the complex trauma group. Trauma types across groups are depicted in Table 1 to gain more insight in types of trauma participants experienced. Age was the only exclusion criteria for the traumatized groups; children had to be aged 8–18 years. Children older than 12 years old and parents with custody had to sign informed consent forms. As the control group was recruited through convenience sampling in an informal network setting, it was compared to both single and complex trauma groups on age and gender composition. Inclusion criteria for children in the control group were no exposure to traumatic events, age between 8 and 18 years old, and a non-clinical score on the CRIES-13 (a posttraumatic stress questionnaire; see under Variables).

**Table 1:** Frequency of type of traumatic experiences across groups and means and standard deviations of age and gender

Type of trauma	Control group (*n* = 40)	Single trauma Group (*n* = *41)*	Complex trauma group (*n* = *38)*
Traffic accident		29	–
Severe bullying		4	3
Maltreatment		2	30
Sexual abuse/assault		2	5
Other		4	–
Mean age (SD)	13.88 (2.50)	14.00 (2.04)	13.03 (2.73)
Female sex (%)	17 (42.50)	24 (58.53)	24 (63.16)

### Variables

#### Executive functions

The Global Executive Composite (GEC) of the Dutch parent version of the Behavior Ratings Inventory Executive Function (BRIEF) was used to measure everyday EF in our participants [32]. The parental questionnaire consists of 75 items. The Behavior Regulation Index (BRI) covers three clinical subscales: Inhibit, Shift, and Emotional Control. The five other subscales, Initiate, Working Memory, Plan/Organize, Organization of Materials, and Monitor, are covered by the Metacognition Index (MI). Statements such as “*he*/*she struggles with starting homework or chores*” and “*he*/*she gets upset very quickly*” are scored on a three-point scale (*1* = *never, 2* = *sometimes, 3* = *often*) and were rated by caregivers. Previous study results have shown that the parent version of the BRIEF, including the GEC, is a reliable and valid instrument of measuring EF in daily life for youth from 5 to 18 years old. The questionnaire shows good psychometric properties (test–retest reliability = 0.86, Cronbach’s *ɑ* = 0.96). Construct and convergent validity of the BRIEF was examined in several large normative samples and found to be satisfactory [16, 32–34]. The instrument was also reliable in our sample with a Cronbach’s alpha of 0.98.

#### Posttraumatic stress

The Dutch version of the Children’s Revised Impact of Events Scale-13 (CRIES-13), a 13-item-questionnaire, was used to measure posttraumatic stress in participants after experiencing a traumatic event [35–37]. The CRIES-13 is a screening questionnaire for youth from 8 to 18 years that assesses the risk for PTSD in youth based on the PTSD criteria of the DSM-IV-TR [38]. The questionnaire has a good construct validity and factor structure [39] and was found to be a valid and reliable screening instrument in a Dutch sample including youth exposed to both single and complex trauma [35]. A score above the cut-off (>30) is associated with an increased risk of PTSD. For example, children and adolescents responded on items as “*Do pictures about it pop into your mind?*” and “*Do you stay away from reminders of it?*” Items were scored on a scale (*0* = *not at all, 1* = *rarely, 3* = *sometimes, 5* = *often*) and were summed for a total score [36]. Three subscales that correspond to the DSM-IV TR criteria of PTSD can be distinguished in this questionnaire: intrusion, avoidance, and arousal. Internal consistency and test–retest reliability of the CRIES-13 is high: *ɑ* = 0.89 and trr = .85 [35]. This was also the case in our sample with a Cronbach’s alpha of 0.90.

### Data analysis

First, we evaluated assumptions for analysis of variance (ANOVA) and mediation analysis. Assumptions of linearity and homoscedasticity were met. The dependent variable, posttraumatic stress symptoms, was not normally distributed. However, ANOVA and mediation analyses are robust against violations of normality [40].

Prior to analyses to test our hypotheses, demographic variables were checked to assess whether or not the three groups differed with regard to age and gender composition. Results of the one-way ANOVA and Chi-square test showed that the three groups did not differ significantly on age (*F* (2,116) = 1.853, *p* = .161) nor gender (*Χ* ^2^ (2) = 3.742, *p* = .154). In other words, age and gender composition of the control, single trauma, and complex trauma groups were similar, and could be excluded as possible confounder variables in further analyses. Age and gender could also be excluded as possible confounder variables in the mediation analyses, because one-way ANOVA and *Χ* ^2^ tests showed no significant correlations between age and EF (*F* (1,118) = 1.753, *p* = .094) and age and posttraumatic stress (*F* (1,118) = 1.156, *p* = .333) nor between gender and EF (*Χ* ^2^ (39) = 40.911, *p* = .387) and gender and posttraumatic stress (*Χ* ^2^ (51) = 57.181, *p* = .256).

**Fig. 2:**
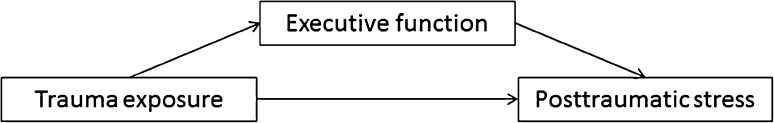
Conceptual mediation model

To investigate the first hypotheses, we analyzed the bivariate links between trauma exposure and EF with Pearson correlations. Second, to investigate group differences in EF between single trauma group, complex trauma group, and control group, a one-way ANOVA was conducted. To investigate the final hypothesis about the mediating role of EF in the relationship between trauma exposure and posttraumatic stress, a mediation analysis with a multi-categorical independent variable (in our case, trauma exposure) was conducted based on the Process Macro for SPSS [41] and an expert tutorial [42]. Process uses ordinal least squares regression analyses for the first two steps of mediation analysis and bootstrap samples for mediator analysis. Process enables the use of multi-categorical independent variable by dummy coding the independent variable. We used an alpha level of 0.05 with bootstrap samples set to 1000 estimates. This analysis is mathematically identical to an analysis of covariance, but also reproduces group means for the mediator and dependent variable. Therefore, it is possible to obtain model, parameter estimates, and model fit statistics that gives us information about how the single trauma group and complex trauma group differ from each other compared to a reference group, in our study participants who are not exposed to a traumatic event [42]. The conceptual mediation model is depicted in Fig. 2. We performed a priori power analyses using G*Power for the first two steps in the mediation analysis: correlation between the independent and dependent variable and correlation between the independent and mediator. A sample of 68 was sufficient for an alpha level of 0.05, a medium effect size (*F* ^2^ = 0.15), and power of 0.80. As the PROCESS macro uses bootstrapping to 1000 estimates to construct confidence intervals, power issues are highly unlikely in the mediation analysis.

## Results

To investigate bivariate links between two groups (single/complex trauma-exposed and control groups) and EFs, we calculated Pearson correlations (see Table 2). Positive significant correlations (*p* < .05) between trauma exposure and EF measures were found. This shows that participants exposed to traumatic events reported more deficits in the global executive composite (GEC), compared to participants in the control group. Supplementary, we investigated bivariate links between the indices metacognition (MI) and behavioral regulation (BRI) and posttraumatic stress symptoms by calculating Pearson correlations (see Table 2). The positive correlations between all indices and subscales of EFs and posttraumatic stress were significant. This shows that more deficits in EF were associated with higher levels of posttraumatic stress symptoms in youth.

**Table 2:** Correlations between trauma exposure, executive function, and posttraumatic stress

	Trauma exposure	Executive function			Posttraumatic stress		
		GEC	BRI	MI	In	Av	Ar
Executive function							
Global executive (GEC)	0.34*						
Behavior regulation (BRI)	0.29*	0.91*					
Metacognition (MI)	0.32*	0.95*	0.77*				
Posttraumatic stress							
Intrusion (In)	0.24*	0.37*	0.37*	0.31*			
Avoidance (Av)	0.41*	0.41*	0.42*	0.35*	0.78*		
Arousal (Ar)	0.45*	0.49*	0.50*	0.43*	0.68*	0.74*	
Total	0.40*	0.47*	0.47*	0.40*	0.87*	0.90*	0.87*

*p < .05

Although results from the correlational analyses indicated a general association between trauma complexity and EF, this did not indicate whether there would be a linear decrease in EF between control, single trauma, and complex trauma groups. Thus, to investigate group differences in EFs, we conducted a one-way ANOVA. Results indicated that the groups differed significantly on the Global Executive Composite (*F* (2,116) = 19.290, *p* = .000, *η* ^*2*^ = 0.25). Table 3 displays mean scores and standard deviations. Post hoc Bonferroni comparisons showed that while the control group did not differ significantly (*p* = .448) from the single trauma group in terms of EF, it did differ significantly from the complex trauma group (*p* < .001). There was also a significant difference between the single trauma group and complex trauma group (*p* < .001). This indicates that participants in the complex group showed more deficits in EF compared to both control group and single trauma group. Additionally, a one-way ANOVA was conducted on the level of posttraumatic stress symptoms across groups. Results indicated that the groups differed significantly on posttraumatic stress symptoms (*F* (2,116) = 19.255, *p* < .001, *η* ^*2*^ = 0.25). Post hoc Bonferroni comparisons showed that the control group did differ significantly from the single trauma group (*p* = .047), and from the complex trauma group (*p* < .001) in terms of posttraumatic stress levels. The difference in posttraumatic stress symptoms was also significant between the single and complex trauma groups (*p* = .001).

**Table 3:** Means and standard deviations of EF and posttraumatic stress in control, single trauma, and complex trauma groups

	Control group		Single trauma group		Complex trauma group		*F*	*p*
	Mean	SD	Mean	SD	Mean	SD		
Executive function								
Global executive	47.10	9.08	50.22	9.18	60.16	10.72	19.29	0.000
Behavior regulation	48.20	9.80	50.15	10.69	60.84	9.95	17.40	0.000
Metacognition	47.15	8.32	49.80	8.12	58.32	10.40	16.39	0.000
Posttraumatic stress								
Intrusion	4.45	4.04	5.51	6.31	9.63	6.02	9.44	0.000
Avoidance	3.10	3.35	6.12	6.77	11.24	5.84	21.57	0.000
Arousal	4.20	3.42	8.14	6.42	13.29	7.11	23.50	0.000
Total	11.75	8.49	20.05	18.16	33.00	17.12	19.26	0.000

The estimated model coefficients to investigate the mediating role of EF in the relationship between trauma exposure and posttraumatic stress are displayed in Table 4. The association between single trauma and EF compared to the control group was not significant. On the other hand, the positive association between complex trauma and EF was significant compared to the control group. In other words, youth in the complex trauma group scored 13.06 points higher on EF (which corresponds with more deficits) compared to the control group. Furthermore, with EF in the model, the positive association of complex trauma with posttraumatic stress remained significant (higher score reflects more posttraumatic stress symptoms). EF had a small, but significant, positive association with posttraumatic stress. The total indirect effect of complex trauma on posttraumatic stress through EF was also significant with a coefficient (*B* = 6.10, boot SE = 2.02, 95% CI 2.25–10.17). This means that there is a genuine, but partial, mediating role for EF in the link between complex trauma and posttraumatic stress.

**Table 4:** Coefficients of PROCESS mediation model

	Executive function (EF)	Posttraumatic stress
	*B* (SE)	*B* (SE)
Model excluding (EF)		
Constant	–	11.75 (1.36)*
Single trauma	–	8.30 (3.18)*
Complex trauma	–	21.25 (3.13)*
Model including (EF)		
Constant	47.10 (1.45)*	**−**10.26 (7.11)
Single trauma	3.12 (2.05)	6.84 (3.05)*
Complex trauma	13.06 (2.28)*	15.15 (3.40)*
Executive function (EF)	–	0.47 (0.15)*

SE’s are bootstrapped SE’s. We used unstandardized B’s in order to interpret regression coefficient easily in comparison with the measurement units

*p < .05

*EF* executive function

### Auxiliary analyses

We performed auxiliary analyses to explore the possible mediating role of three subscales of the BRIEF, namely inhibition, flexibility, and working memory. Separate mediation analyses showed the same patterns as the previous mediation model with total EF as a mediator. The total indirect effects of complex trauma on posttraumatic stress through inhibition (*B* = 4.54, boot SE = 1.81, 95% CI 1.62–9.41), through working memory (*B* = 4.97, boot SE = 1.94, 95% CI 1.54–9.44), and through flexibility (*B* = 5.08, boot SE = 1.82, 95% CI 2.19–9.48) were significant.

## Discussion

The results of the present analyses indicate that, indeed, trauma-exposed youth experience more deficits in EF compared to participants who did not experience traumatic events. More specifically, our results indicate quite clearly that children and adolescents exposed to complex trauma experienced more deficits in EF than youth exposed to a single traumatic event. In addition, our results revealed that EF partially mediates the relationship between complex trauma exposure and posttraumatic stress symptoms. That is, participants exposed to complex trauma had more deficits in EF, and this in turn was associated with more posttraumatic stress symptoms.

In line with our first hypothesis and previous research, trauma exposure was associated with more deficits in EF compared to youth that did not experience traumatic events [20, 23]. Complexly traumatized youth in our sample showed more deficits in EF compared to youth exposed to single trauma or non-traumatized children. We also found that complexly traumatized children and adolescents had a subclinical mean score on the EF measure; their reported EF difficulties should be taken into account by a (neuro) psychologist for further assessment. Additionally, we found that youth exposed to single trauma did not have more deficits in EF than participants in the control group. The cumulative risk model of psychopathology [43] and the cumulative stressors model [44] help explain these findings. Children’s developing brains might be more resilient against exposure to one severe traumatic event in terms of EF compared to exposure to complex trauma, and therefore to chronic stress [6].

Besides the model of cumulative stressors, another plausible explanation for our findings could be the nature of trauma exposure. Generally, complex trauma exposure has an interpersonal character, while single trauma exposure mostly includes events such as traffic accidents or earthquakes [1]. It might be that emotionally charged traumas such as child sexual abuse or child maltreatment have more severe effects on the developing brain than non-interpersonal trauma such as earthquakes or traffic accidents. This could be an alternative explanation as the majority of the single trauma group was exposed to traffic accidents. Therefore, EF could be more affected by complex trauma than by single trauma [6, 24]. In this case, it is not the accumulation of traumatic events that cause executive dysfunction, and in turn posttraumatic stress, but rather the emotional character of the traumatic events.

The mediation analysis showed that EF is a partial mediator in the relationship between complex trauma and posttraumatic stress symptoms. Reasonably, trauma exposure played the most important role in predicting levels of posttraumatic stress symptoms. From a neuropsychological and developmental perspective [6], it might be that youth exposed to complex trauma show more severe posttraumatic stress symptoms through their deficits in EF. Due to problems with inhibition, fear responses and hypervigilance symptoms arise. Subsequently, because these children and adolescents cannot inhibit the fear response on triggering stimuli, they develop an avoidant coping strategy [23].

There are several limitations to our study. The most prominent limitation is our cross-sectional research design. It prohibits us from drawing causal conclusions based on the analyses. Although it is logical that posttraumatic stress follows trauma exposure as it is within the definition, it could be possible that youth with EF deficits are at higher risk for traumatic exposures due to parental conflicts or interpersonal problems [45]. It could also be that children with EF deficits are more sensitive to develop posttraumatic stress symptoms. To the best of our knowledge, there are no prospective studies that measured the predictive relationship between EF and PTSD. Therefore, as an experimental design is not feasible within this research context, the next step should be to employ longitudinal research to investigate the developmental trajectory of posttraumatic stress in relation to EF in youth. In addition, prior to analysis, group composition was only tested for the variables age and gender. More demographic variables such as socio-economic status, ethnicity, and IQ should be included to exclude possible confounding variables. As timing of trauma could be an important factor in the development of PTSD, future studies should use a longitudinal approach to assess this relationship. Third, the use of questionnaires to assess posttraumatic stress and EF is limited. More information from specific EF tasks, related to inhibition, flexibility, and working memory, could give researchers more insight in EF deficits in children exposed to traumatic events.

### Future research

We suggest that the strong association between EFs and posttraumatic stress demonstrates that complex trauma exposure is associated with a broader range of problems in youth. This is aligned with earlier research findings that complexly traumatized children and adolescents, compared to youth exposed to single trauma, show more developmental problems besides posttraumatic stress symptoms such as intrusion, avoidance, and arousal [24, 46, 47]. Many propositions have been made for the concept of developmental trauma disorder after exposure to single or complex trauma [6, 24]. Although we cannot draw conclusions about the etiology of posttraumatic stress through EF, our findings may give guidelines to investigate a broader range of consequences following exposure to complex trauma. Therefore, we strongly recommend research at neurological level (brain imaging research) and neuropsychological level to gain more insight in the possible mediating role of EF in posttraumatic stress.

Our findings can have important implications for clinical practice. When deficits in EF are acknowledged as additional consequences of complex trauma exposure or as a mediator in the development of posttraumatic stress, trauma therapy and prevention can be adjusted or expanded. For example, cognitive training programs might improve EF also in traumatized youth. Training could diminish the negative consequences on children’s and adolescents’ academic and social development. In turn, it could prevent youth from developing posttraumatic stress and thereby reduce or alleviate adverse consequences on their development. Additionally, combining cognitive training and trauma therapy might enable them to benefit more or faster from techniques learned in psychotherapy.

In conclusion, we found strong associations between complex trauma, EF, and posttraumatic stress in youth with strong indications for a partial mediating role of EF on the development of posttraumatic stress. This means that complexly traumatized youth show more deficits in EF, which is associated with higher levels of posttraumatic stress. Our research findings should be replicated longitudinally to give definitive answers to the question how trauma exposure, EF, and posttraumatic stress are associated in children and adolescents. This may yield more effective clinical practice that is able to tackle the negative consequences of trauma exposure in children’s development.
